# Detection of Inter-Lineage Natural Recombination in Avian Paramyxovirus Serotype 1 Using Simplified Deep Sequencing Platform

**DOI:** 10.3389/fmicb.2016.01907

**Published:** 2016-11-30

**Authors:** Dilan A. Satharasinghe, Kavitha Murulitharan, Sheau W. Tan, Swee K. Yeap, Muhammad Munir, Aini Ideris, Abdul R. Omar

**Affiliations:** ^1^Laboratory of Vaccine and Immunotherapeutic, Institute of Bioscience, Universiti Putra MalaysiaSerdang, Malaysia; ^2^Faculty of Veterinary Medicine and Animal Science, University of PeradeniyaPeradeniya, Sri Lanka; ^3^Infection and Innate Immunity Research Group, Avian Viral Diseases, The Pirbright InstituteSurrey, UK; ^4^Faculty of Veterinary Medicine, Universiti Putra MalaysiaSerdang, Malaysia

**Keywords:** Newcastle disease virus, avian paramyxovirus 1, next-generation sequencing, phylogenetic analysis, recombination

## Abstract

Newcastle disease virus (NDV) is a prototype member of avian paramyxovirus serotype 1 (APMV-1), which causes severe and contagious disease in the commercial poultry and wild birds. Despite extensive vaccination programs and other control measures, the disease remains endemic around the globe especially in Asia, Africa, and the Middle East. Being a single serotype, genotype II based vaccines remained most acceptable means of immunization. However, the evidence is emerging on failures of vaccines mainly due to evolving nature of the virus and higher genetic gaps between vaccine and field strains of APMV-1. Most of the epidemiological and genetic characterizations of APMVs are based on conventional methods, which are prone to mask the diverse population of viruses in complex samples. In this study, we report the application of a simple, robust, and less resource-demanding methodology for the whole genome sequencing of NDV, using next-generation sequencing (NGS) on the Illumina MiSeq platform. Using this platform, we sequenced full genomes of five virulent Malaysian NDV strains collected during 2004–2013. All isolates clustered within highly prevalent lineage 5 (specifically in lineage 5a); however, a significantly greater genetic divergence was observed in isolates collected from 2004 to 2011. Interestingly, genetic characterization of one isolate collected in 2013 (IBS025/13) shown natural recombination between lineage 2 and lineage 5. In the event of recombination, the isolate (IBS025/13) carried nucleocapsid protein consist of 55–1801 nucleotides (nts) and near-complete phosphoprotein (1804–3254 nts) genes of lineage 2 whereas surface glycoproteins (fusion, hemagglutinin-neuraminidase) and large polymerase of lineage 5. Additionally, the recombinant virus has a genome size of 15,186 nts which is characteristics for the old genotypes I–IV isolated from 1930 to 1960. Taken together, we report the occurrence of a natural recombination in circulating strains of NDV in commercial poultry using NGS methodology. These findings will not only highlight the potential of RNA viruses to evolve but also to consider the application of NGS in revealing the genetic diversity of these viruses in clinical materials. Factors that drive these evolutionary events and subsequent impact of these divergences on clinical outcome of the disease warrant future investigations.

## Introduction

Newcastle disease virus (NDV) is a type species of an avian paramyxovirus serotype 1 (APMV-(1), which belongs to genus *Avulavirus* in the family *Paramyxoviridae*. The virus carry negative-sense, single-stranded, and non-segmented RNA genome, which encodes for at least six structural proteins, including nucleocapsid protein (NP), phosphoprotein (P), matrix protein (M), fusion protein (F), haemagglutinin-neuraminidase (HN), and large protein (L) (Chambers et al., 1986). Through the RNA editing of the P gene, one accessory non-structural protein (known as V) is synthesized, and probability a second protein named W is also produced (Locke et al., 2000). The HN and F proteins are surface glycoproteins that determine the virus neutralization and protection. The NP, P, and L proteins encapsidate the viral nucleic acid; the M protein lined the virus envelope and V protein interfere with interferon (IFN) responses (Dortmans et al., 2011; Zenglei and Liu, 2015).

Depending on the pathogenicity in chicken, NDV strains are classified into highly virulent (velogenic), intermediate (mesogenic), and avirulent (lentogenic) strains (Alexander, 1997). The amino acid sequence of the F protein cleavage site has been used to determine the pathogenicity of NDV (Nagai et al., 1976). Generally, the sequence of the F protein cleavage site in mesogenic and velogenic strains of NDV consists of a polybasic cleavage site (R/K) RQ(R/K) R↓F, which is readily recognized by ubiquitous furin, an intracellular protease abundant in several cells and tissues, and consequently cleaves in many tissues resulting in systematic infection. In contrast, initiation of viral infection by cleavage of the F protein in lentogenic strains is restricted to respiratory and enteric systems and thus limiting the infections in these organs (Collins et al., 1993).

Although all NDV's strains are grouped within APMV-1, yet viruses show significant genetic diversity (Alexander, 1997; Aldous et al., 2003; Kim et al., 2007a). Without any incongruity, there are two methods which are currently being used to classify APMV-1 strains into different clusters. Aldous et al. (2003) have divided APMV-1 into six lineages and 13 sub-lineages, which later included three sub-lineages (Aldous et al., 2003; Snoeck et al., 2009). According to the second system, APMV-1 has been divided into two main categories which are represented as class I and class II. The class I is also subdivided into nine genotypes while the class II is subdivided into at least 13 genotypes (Ballagi-Pordány et al., 1996; Czeglédi et al., 2006; Kim et al., 2007b). In general, there is just slight dissimilarity that exists amid the two systems (Miller et al., 2010) in which lineage 6 comprised class I viruses and lineage 1, 2, 4, and 5 consisted of viruses belonging to genotype I, II, VI, and VII of class II, respectively. Furthermore, highly diverse lineage 3 comprised of genotype III, IV, V, and VIII of class II viruses (Miller et al., 2010). Several previous studies have shown that genotype V, VI, and VII of class II viruses were the most prevalent viruses presently circulating globally. Out of these, lineage 5 or genotype VII of class II viruses were the most predominantly isolated strains of NDV from Asia, Africa, Middle East, and South America (Khan et al., 2010; Miller et al., 2010; Munir et al., 2012; Perozo et al., 2012). A recent study has classified NDV strains into several new genotypes based on the mean inter-populationary evolutionary distance of 10% as the cutoff value (Diel et al., 2012). Based on that study, viruses in class I formed a single genotype, while class II viruses were separated into 15 genotypes including 10 earlier established genotypes (I - IX and XI) and five new genotypes (X, XII, XIII, XIV, and XV). The subsequent study introduced two additional genotypes into this classification system (XVII and XVIII) from NDV outbreaks occurring in the Asia, west, and central Africa (Munir et al., 2012; Snoeck et al., 2013). Taken together, none of the classification systems represent a clear lineage/genotype-specific disease potential, geographical distribution or host specifications.

Similar to other RNA's viruses, NDV holds potential for evolution (Miller et al., 2010). However, limited information is available that clearly defines events of evolution, recombination, and possible gained pathogenicities of NDV strains in avian hosts. This lack of information is partly due to (i) mainly NDVs are characterized based on the partial sequence of F or HN genes which are insufficient to identify recombination (Song et al., 2011), (ii) full genetic characterized of majority of APMVs is performed using conventional methodologies (PCRs and Sanger sequencing) which is not only error prone but also less capable to identify poorly presented virus population in the complex samples, and (iii) initial clinical material appeared to be insufficient for amplification of full genome in conventional PCRs. This requires propagation of viruses in chicken eggs, and this process may favor the replication of fitter viruses and masking the recombined strains that by any means are lower in population. Despite these shortcomings, recombination events, and evolution diversities have been reported in the NDV (Qin et al., 2008; Chong et al., 2010). Previously, Han et al. (2008) have reported a natural recombination NDV isolate named cockatoo/Indonesia/14698/90 (AY562985) with anonymous major parental lineage and two minor parental-like lineages derived from vaccine lineage and anhinga/U.S. (Fl)/44083/93 lineage, in that order (Han et al., 2008).

One of the driving forces for recombination and increasing pathogenic potential of viruses is the immune pressure imposed by the extensive vaccination (Read et al., 2015). For NDV, the primary means of disease control has been the employment of exhaustive vaccination program in both layer and broiler poultry sectors. Despite mass vaccination being practiced, NDV outbreaks have been reported throughout the world and have led to substantial economic losses to the industry over the years (Miller et al., 2010). This escaped protection has been linked to the partial protection by the current vaccines and persistent virus shedding in the immunized birds (Xiao et al., 2012; Samuel et al., 2013).

In Malaysia, an intensive vaccination program has been placed, however, the disease is continuously emerging, and the impact is enormous (Yusoff and Tan, 2001; WAHID, 2015). In this regards, we have recently characterized several isolates of NDV from various states of Malaysia from 2004 to 2005 (Tan et al., 2010). This characterization was based on the partial F gene sequences which limit the full genetic characterization and detection of recombination events. Here, we optimized a simple, robust and less resource-demanding methodology for the whole genome sequencing of APMVs using next-generation sequencing (NGS) technology. Using this protocol, we generated unbiased consensus-level full genomes of five APMVs strains which were obtained from clinical outbreaks during 2004–2013 in Malaysia. Phylodynamics and evolutionary analysis revealed recombination between lineage 2 and 5 and shown features that are unique for this lineage/genotype of APMV-1. The presented results are crucial to establishing bases for the vaccine-induced immune pressures, vaccine failure, and potential of APMVs to evolve for higher pathogenicity.

## Materials and methods

### Samples collection

Five NDV isolates were obtained from individual outbreaks in the commercial poultry farms that were vaccinated for Newcastle disease (ND) located in different states in Malaysia (Table 1). Two isolates, IBS002/11 and IBS005/11, were isolated in 2011 while other three isolates, MB128/04, MB 076/05, and IBS025/13, were isolated in 2004, 2005, and 2013, respectively. Apart from IBS025/13, all samples were detected positive previously using reverse transcriptase PCR (RT-PCR) which is based on the partial F gene (data not shown). In addition, these isolates have been characterized in the past as genotype VII NDV isolates which are based on the partial F gene sequencing (Berhanu et al., 2010; Tan et al., 2010; Roohani et al., 2015).

**Table 1:** **Clinical description and vaccination history of Malaysian NDV isolates used in this study**.

**NDV isolate**	**Year**	**Origin**	**Farm type**	**Case history**	**Vaccination history**	**References**
MB128/04	2004	Selangor	Broiler	Not available	Not available	Berhanu et al., 2010
MB076/05	2005	Sabah	Broiler	Depression, marked conjunctivitis, anorexia, and facial edema	Not available	Berhanu et al., 2010
IBS002/11	2011	Johor	Broiler	The infection started at day 14 with 5% mortality. Bird showed signs of coughing, sneezing, and torticollis	At day 8 and 14 with live ND+IB	Roohani et al., 2015
IBS005/11	2011	Penang	Broiler	The infection started at day 29 with 5% mortality. Depression and greenish diarrhea, respiratory rales	At day 2, 8, and 12 with ND+IB	Roohani et al., 2015
IBS025/13	2013	Penang	Broiler	The infection started at day 18 with 4% mortality. Bird showed signs of coughing, sneezing, greenish diarrhea and torticollis	At day 14 with ND+IB	This study

ND, Newcastle disease; IB, Infectious bronchitis.

### Virus specimens

All NDVs used in this study were triple plaque-purified on chicken embryo fibroblast (DF1 cells) and propagated by inoculating into 9 days-old specific-pathogen-free (SPF) embryonated chicken eggs and stored in liquid nitrogen. Virus stocks of the selected NDV isolates were thawed and propagated in the allantoic cavity of 9 days old SPF embryonated chicken eggs according to European Community Directive 92/66/EC (CEC, 1992) and identified using hemagglutination (HA) test. Allantoic fluids from the sample showing high HA titers more than 2^7^ were divided into working stocks and stored at −20°C. These allantoic fluids were used as working stocks for pathogenicity assessments and sequencing.

### RNA extraction and quality control

Genomic viral RNA was extracted from the infected allantoic fluid of SPF eggs by using TRIzol® Reagent (Invitrogen, USA) according to the manufacturer's instructions. After air drying, the RNA pellet was resuspended in 40 μL nuclease-free water (Ambion, USA). The quantity and purity of the extracted RNA were checked by Eppendorf BioSpectrometer® subsequently, and was then stored at −80°C for further analysis.

### Designing of primers

Based on the complete genome of NDV strain chicken/Banjarmasin/010/10 (HQ697254.1), five pairs of primer sets (Table 2) were designed to amplify the whole genome of five Malaysian NDV isolates. The full genome of NDV was divided into 5 fragments encompassing hypervariable regions flanked by more conserved sequence regions. The fragments that were identified with the most conserved regions were subjected to NCBI Primer 3 and Primer-BLAST tool and five diverse primer pairs were designed. An additional primer pair was designed for the third fragment, which consists of the highly variable regions.

**Table 2:** **Primers used to amplify the complete genome of lineage 5 NDV**.

**Fragment**	**Location**	**Forward primer sequence (5′ → 3′)**	**Reverse primer sequence (5′ → 3′)**	**Size**
1	1–2792	ACCAAACAGAGAATCTGTGAGGTAC	TCGGGCTACTGCTCGGAGAT	2792
2	2541–5926	GTCCAGCTACCTGCCGACTT	GCCTGTCACGATGACTTGAGA	3386
3^*^	5673–9156	CTGAAGGCGCACTCACTACG	AGATGTGCCGCCGTAGAAGAT	3484
	4258–8657	CCTGCGGAGTGTGAAAGTCA	GGAGCACTCCGGTTATCTTGG	4400
4	8644–12,183	AACCGGAGTGCTCCATCCC	AGATTCCCAGCTGTGGGCAG	3540
5	11,745–15,192	CCCGGAACAGAAGCTGGTCA	ACCAAACAGAGATTTGGTGAACGAC	3448

* An additional primer was designed for fragment 3 which was located in the hypervariable region of the genome. Primer locations are based on NDV strain chicken/Banjarmasin/010/10 (HQ697254.1).

### cDNA synthesis

First-strand cDNA synthesis (reverse transcription) was performed using MMLV Reverse Transcriptase First-Strand cDNA synthesis kit (Epicentre, USA). Briefly, the following components were combined on ice: 6.5 μL of RNase-Free Water, 5 μL of viral RNA sample and 1.5 μL of fragment 1 forward primer (Table 2) for a 12.5 μL total reaction volume, incubated at 65°C for 2 min with heated lid and chilled on ice for 1 min. A second reagent mix of 2 μL MMLV RT 10 × Reaction Buffer, 2 μL 100 mM DTT, 2 μL dNTP PreMix, 0.5 μL RiboGuard RNase Inhibitor and 1 μL MMLV Reverse Transcriptase were added to each first-strand cDNA synthesis reaction and mixed gently on the ice. The reaction was incubated at 37°C for 60 min followed by heating at 85°C for 5 min and chilled on ice for at least 1 min. The cDNA was briefly centrifuged and immediately used or stored at −80°C for future analysis.

### Synthesis of double strand DNA

Based on the manufacturer's instructions of using KAPA HiFi Hot Start Ready Mix (KAPA Biosystems, USA) kit, the cDNA was used to synthesize a double strand DNA. Briefly, 3 μL of cDNA was combined with 12.5 μL of 2 × KAPA HiFi Hot Start Ready Mix, 0.9 μL of 10 μM forward primer and reverse primer up to 25 μL volume with PCR-grade water. The thermal cycling protocol for the PCR was included with an initial denaturation at 95°C for 3 min followed by a sequence of 30 cycles, which comprised denaturation at 98°C for 20 s, annealing at 60°C for 20 s, extension at 72°C for 2 min and final extension hold at 72°C for 5 min. The PCR product was detected in 0.8% agarose gel electrophoresis and purified using MEGAquickspin™ Total Fragment DNA Purification Kit (iNtRON Biotechnology, South Korea) as per manufacturer's instructions.

### Next-generation sequencing library preparation and sequencing

Each DNA originated from the isolates was quantified by using Qubit dsDNA HS Assay Kit (Invitrogen, USA) and then normalized to 0.2 ng/μL. A total of 1 ng of DNA was subjected to library preparation using Nextera XT DNA Sample Prep Kit (Illumina Inc., San Diego, CA, USA) by following the manufacturer protocol. Briefly, the DNA was tagmented (fragmented and tagged) by the Nextera XT transposome. The tagmented DNA was used as template in a 50 μl of PCR with 12 cycles and processed as outlined in the Nextera XT protocol. Additionally, AMPureXP beads (Beckman Coulter Inc., Fullerton, CA, USA) was applied to purify the amplified DNA.

After PCR clean up, DNA fragment size and library concentration was analyzed by using 2100 Bioanalyzer (Agilent Technologies, USA) and Library Quantification Kit (KAPA Biosystems, USA). Afterward, DNA libraries were normalized to 4 nM and libraries with unique indexes were pooled in equal volumes. Pooled libraries were denatured and diluted with 0.2 N NaOH and pre-chilled Hybridization Buffer (HT1) to produce a denatured 12 pM library in 1 mM NaOH solution. The final library was sequenced using MiSeq (Illumina Inc., San Diego, CA, USA) with the read length of 2 × 150 bp.

### Analysis of the data

The overlapping paired-end reads were filtered on the Phred quality score (Q30) and imported to CLC Genomics Workbench software version 7.5.1 (CLC bio, Aarhus, Denmark) for adapter trimming and *de novo* assembly of the paired-end reads to contigs. The contigs were then subjected to BLASTN at NCBI and based on the highest sequence similarity and lowest *E*-value, reference genomes were determined. Low coverage contigs were excluded and when necessary, partial but overlapping contigs were combined. Final consensus having more than 99% coverage to full genome sequences were then examined for appropriate assembly depending on the length and the presence of the expected intact NDV open reading frames.

### Confirmation using sanger sequencing

Two gaps were observed in the consensus of IBS005/11 at the nucleotide positions of 1708–1743 and 1821–1882. Forward primer sequence 5′-GCCATCCCAAGACAACGACA-3′ aligning nucleotide position of consensus at 1555 and the reverse primer sequence 5′- CCCTGGGCCGTTATTATGCT-3′ aligning position of IBS005/11 consensus at 1956 were designed by using NCBI Primer 3 software to close the gap. The PCR product was observed in 1.5% agarose gel. Similarly, 7 nucleotides gap was observed in the consensus of MB128/04 at the nucleotide positions 1698–1708. This gap was closed using the same forward and the reverse primer sequences earlier mentioned. Additionally, the recombination region (1–2766 bp) including the 6 nucleotides deletion at the position 1647 of 5′ NCR of the NP gene was validated twice by sequencing the fragment 1 (Table 2) of the IBS025/13 NDV isolate by primer walking technology.

The cDNA template was synthesized as previously described and PCR was performed according to PCR conditions mentioned above. Both PCR products were purified with MEGAquickspin™ Total Fragment DNA Purification Kit (iNtRON Biotechnology, South Korea) as per manufacturer's instructions. Sanger sequencing was performed from both directions in order to close the gaps and confirmation of recombination region in the IBS 025/13 isolate. Finally, the obtained sequences were aligned with the consensus by using MEGA6 software (Tamura et al., 2013).

### Amplification of the 3′- and 5′- terminal ends of the viral RNA

To determine the leader and trailer at the 3′- and 5′- terminal ends of the viral RNA, rapid amplification of cDNA ends (RACE) was performed as described earlier on de Leeuw and Peeters (1999). The 5′ end primer L-cDNA (5′- AAGTCACAATACTGGGTCTCAG -3′), which was designed from L gene of genotype VII NDV strain, chicken/Banjarmasin/010/10 (HQ697254.1) was used to generate single-stranded cDNA as described above. The T4 RNA ligase (New England Biolabs Inc, USA) was then used to ligate the generated single-stranded cDNA so as to anchor primer (5′- CACGAATTCACTATCGATTCTGGATCCTTC-3′). One micro liter of ligation mixture was used for PCR with KAPA HiFiHotStartReadyMix (KAPA Biosystems, USA) in accordance with the manufacturer's instructions. The primers used were anchor-complementary (5′-GGATCCAGAAT-CGATAGTGAATTCG-3′) and L-PCR (5′-CAGCCAAGGGAT-ATTACAGTAACT-3′). The PCR consisted of 30 cycles of 1 min at 98°C, 20 s at 60°C and 20 s at 72°C. The PCR products were then cloned into pJET 1.2 blunt/cloning vectors (Thermo Fisher Scientific, Inc, USA) and verified by sequencing. The anchor primer was ligated to the 3′ prime end of genomic RNA with T4 RNA ligase as described above in order to determine the sequence of the 3′-terminal ends. Then, the anchor-complementary primer was used to generate the cDNA. Hence, anchor-complementary and NP-PCR primers were used to conduct PCR. Primer (5′-GGAGCTGCTCGTATTCGTC-3′) was used to generate fragments for strains MB076/05, IBS002/11, IBS005/11, and MB128/04. Meanwhile, NP-PCR primer (5′- CGAGGAGCTGTTCGTACTCATCAA-3′) was used for strain IBS025/13. The PCR conditions were the same as have been described above.

### Recombination among NDV sequences

SimPlot program (Ray, 2003) was used to identify putative recombination breakpoints in the sequenced whole genome of NDV isolates and to identify sequences possibly originated from a recombination. The program is based on a sliding window process and consists of a way of graphically displaying the coherence of the sequence relationship over the entire length of a set of aligned homologous sequences. The window width and the step size were set to 200 bp (or 500 bp) and 20 bp, respectively. In addition, Recombinant Program v4.56 (RDP4) was used for the detection of recombination events, likely parental isolates of recombinants and recombinant breakpoints. Various methods such as RDP, GENECONV, Chimaera, MaxChi, BOOTSCAN, and SISCAN with default settings (Martin et al., 2005) were used by the RDP4 program.

### Phylogenetic and pairwise sequence comparisons (PASC) analysis

The nomenclature of the NDV isolates in this study was based on genotypes (Ballagi-Pordány et al., 1996; Czeglédi et al., 2006) and lineages (Aldous et al., 2003; Snoeck et al., 2009; Munir et al., 2012). CLUSTALW (Thompson et al., 1997) was used to align all the NDV sequences while MEGA6 software was used to analyze all the phylogenetic. The model with lowest BIC (Bayesian Information Criterion) value was selected as the most suitable model for phylogenetic analysis (Tamura et al., 2013). The reliability of the lineages defined for NDV was determined by Pair-Wise Sequence Comparisons (PASC) using selected NDV strains available in GenBank representing all lineages except lineage 6 and Malaysian isolates. All reference isolates used in this study were named first with accession number followed by country of origin, respected lineage and year of isolation. Also, mean distances among and within lineages were calculated using PASC in MEGA6 software.

### Ethics statement

The animals used for this research were kept in the house and animal care procedures were conducted according to the local animal welfare regulations and EU directive (EU Directive on the protection of animals used for scientific purposes 2010/63/EU) (EU, 2010) under bio-safety level (BSL2) enhanced experimental animal facility at the Faculty of Veterinary Medicine, UPM. The animal experimental protocol for Intracerebral Pathogenicity Index (ICPI) study was approved by the Institutional of Animal Care and Use Committee (IACUC), Faculty of Veterinary Medicine, UPM (reference number UPM/IACUC/AUP-R028/2013); the local animal care authority. Animals were monitored for a minimum of three times per day by a qualified and registered veterinarian to ensure animal welfare and health. The end point of all these *in vivo* animal experiments was death; although, a humane endpoint was pre-defined in the protocol and applied to prevent any pain, distress or suffering. The humane endpoint was decided for chicks used for ICPI study manifested terminal clinical signs. Animals showing terminal signs including anorexia and paralysis were sacrificed by cervical dislocation under sedation in accordance with standard guidelines (EU, 2010; Leary et al., 2013).

### Intracerebral pathogenicity index assessment in SPF chickens

Pathogenicity of NDV isolates was assessed by using a standard ICPI test as earlier stated (OIE, 2012). Briefly, 50 μl of allantoic fluid of each NDV isolate with an HA titer more than 16 HA units/50 μl and diluted 10-fold in PBS without antibiotics were inoculated intracerebrally to 1-day-old chicks (*n* = 10). Ten chicks were kept as uninoculated control. All the chicks were being monitored at least three times per day for an 8-day period of observation. The NDV isolates that scored an ICPI > 1.5 were identified as velogenic strains, <0.7 as lentogenic and those with intermediate ICPI values were considered as mesogenic strains (OIE, 2012).

## Results

### Whole genome sequencing of NDV

The present study proposes a pipeline to generate consensus level sequences with more than 99% coverage of the full genome. The consensus level genome sequences of five NDV isolates belonging to genotype VII were generated for the first time in Malaysia. The whole genome sequences of these NDV isolates were deposited in the GenBank and are available under the following accession numbers: KR074407 for MB128/04, KR074406 for MB076/05, KR074404 for IBS002/11, KR074405 for IBS005/11 and IBS025/13 for KT355595.

Summary of the genomic features of the Malaysian isolates, including starting and ending positions of each gene, intergenic, and coding regions, represented in Tables 3, 4. The genome length of 4 NDV isolates (MB128/04, MB076/05, IBS002/11, and IBS005/11) and IBS025/13 isolate was calculated to be 15,192 nts and 15,186 nts respectively, which putatively consisted of 6 different genes with each gene containing a single or multiple open reading frames (ORFs) as indicated in Tables 3, 4.

**Table 3:** **Summary of the genome organization and the predicted protein length of the sequenced MB128/04, MB076/05, IBS002/11, and IBS005/11 isolates**.

**Gene**	**Gene start position (nt)**	**3′UTR length (nt)**	**Coding sequence positions (nt)**	**5′UTR length (nt)**	**Gene end position (nt)**	**Intergenic region length (nt)**	**Gene length (nt)**	**Protein length (aa)**
NP	56–65	66	122–1591	217	1799–1808	1	1753	489
P	1810–1819	83	1893–3080	180	3251–3260	1	1451	395
M	3262–3271	34	3296–4390	112	4493–4502	1	1241	364
F	4504–4513	46	4550–6211	84	6284–6295	31	1792	553
HN	6327–6336	91	6418–8133	195	8319–8328	47	2002	571
L	8376–8385	11	8387–15,001	77	15,069–15,078	–	6703	2204

UTR, untranslated region.

**Table 4:** **Summary of the genome organization and predicted protein lengths in the sequenced IBS025/13 isolate**.

**Gene**	**Gene start position (nt)**	**3′UTR length (nt)**	**Coding sequence positions**	**5′UTR length (nt)**	**Gene end position (nt)**	**Intergenic region length (nt)**	**Gene length (nt)**	**Protein length (aa)**
NP	56–65	66	122–1591	210	1791–1801	2	1746	489
P	1804–1813	83	1887–3074	180	3245–3254	1	1451	395
M	3256–3265	34	3290–4384	112	4487–4496	1	1241	364
F	4498–4507	46	4544–6205	84	6280–6289	31	1792	553
HN	6321–6330	91	6412–8127	195	8313–8322	47	2002	571
L	8370–8379	11	8381–14,995	77	15,063–15,072	–	6703	2204

UTR, untranslated region.

### Biological and molecular pathogenicity assessment

The isolates in the current study exhibited ICPI values more than 1.5 and the F protein cleavage site sequences exhibited ^112^R/K-R-Q-R/K-R↓F^117^ motif similar to mesogenic and velogenic NDV strains, which is another characteristic considered by the World Organization for Animal Health (OIE) to assess the pathogenicity (OIE, 2012) (Table 5).

**Table 5:** **Biological and molecular characterization of NDV isolates**.

**NDV isolate**	**F cleavage site**	**ICPI**
MB128/04	^112^RRQKR↓F^117^	1.68
MB076/05	^112^RRQKR↓F^117^	1.84
IBS002/11	^112^RRRKR↓F^117^	1.76
IBS005/11	^112^RRRKR↓F^117^	1.71
IBS025/13	^112^RRQKR↓F^117^	1.63

### Phylogenetic and pairwise sequence comparison (PASC) analysis of NDV isolates

**Figure 1:**
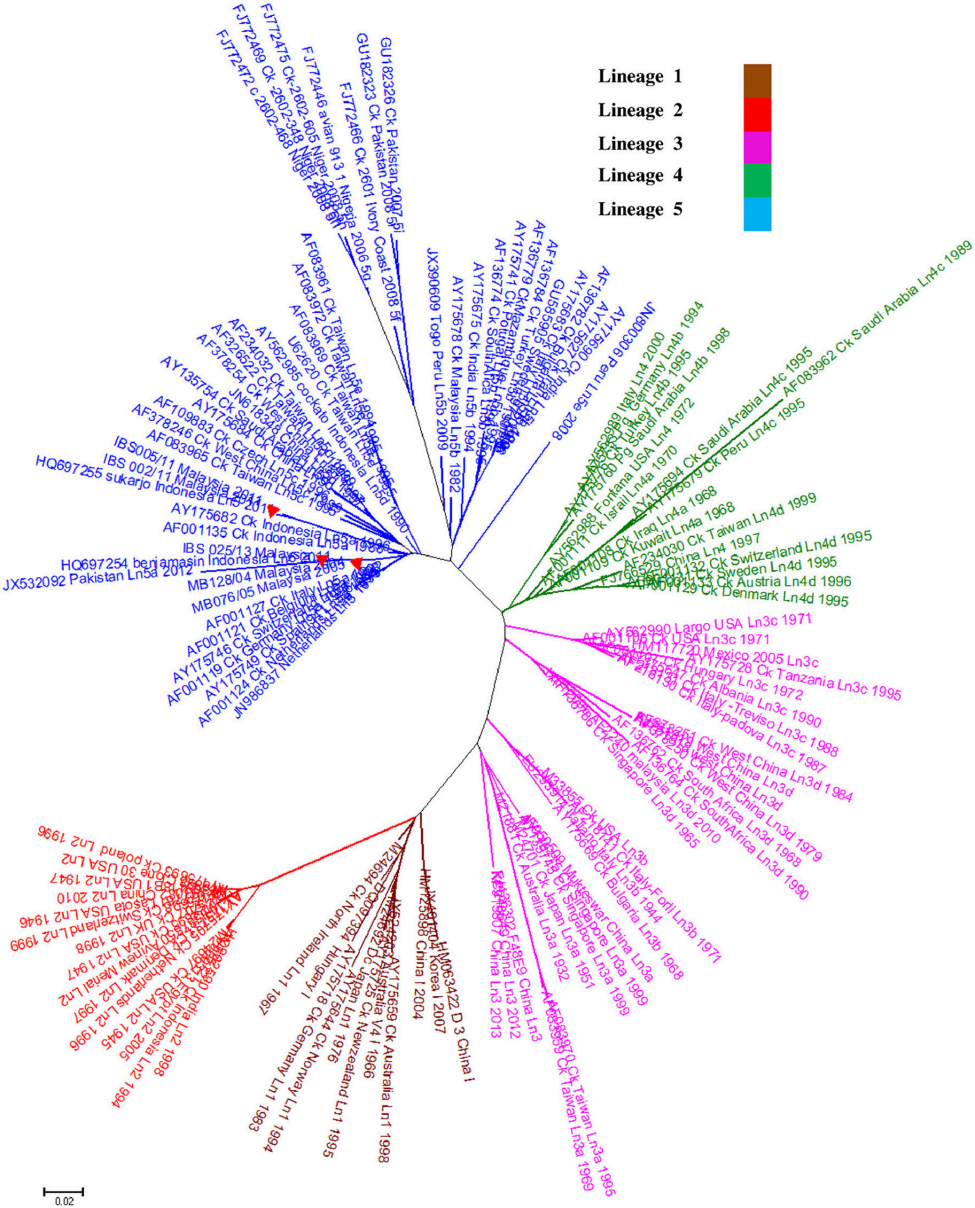
**A phylogenetic analysis of the Newcastle disease viruses; Phylogenetic relationship among 120 published NDV isolates and 5 Malaysian isolates based on F gene nucleotide sequences between 47 and 422 positions**. The sequences used were obtained from GenBank. As identified by Aldous et al. (2003), Class II viruses representing lineages 1–5 (*n* = 120) and Malaysian isolates from 2004 to 2013 (*n* = 5) identified with 373 base pair region encoding the amino terminal end of the fusion protein. Tree construction was done using the Neighbor-Joining method with the maximum composite likelihood substitution model after 1000 bootstrap replication by using MEGA 6. Lineage 6 of APMV-1 was excluded in the analysis. Isolates presented in this study was marked with a red triangle (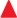).

**Figure 2:**
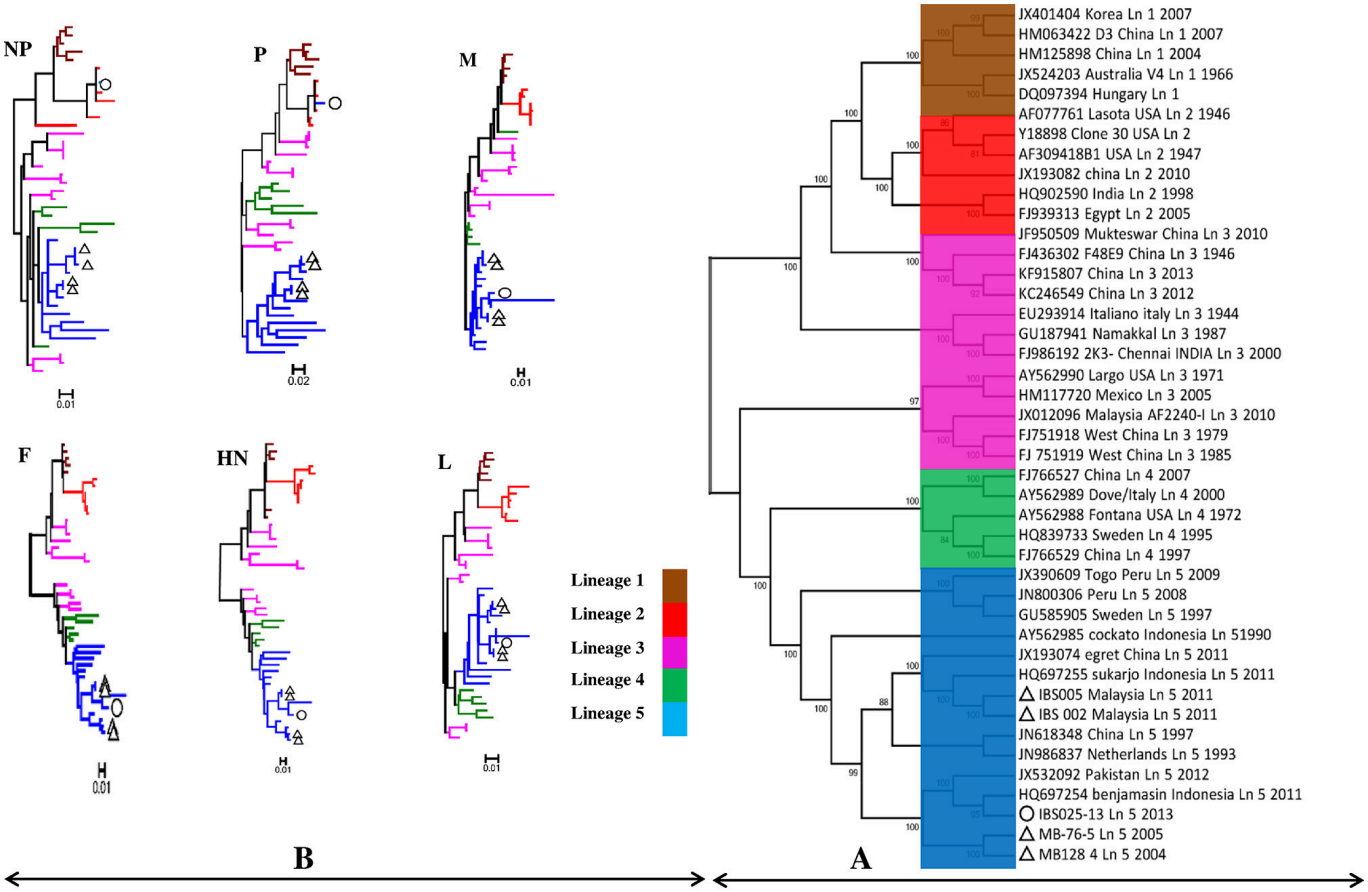
**The maximum likelihood phylogenetic trees constructed by using 5 Malaysian NDV isolates. (A)** The phylogenetic tree based on nucleotide sequence of complete genome compared with 38 representative complete genomes of lineage 1–5 belongs to APMV-1. **(B)** The phylogenetic trees based on amino acids sequence of NP, P, M, F, HN, and L proteins compared with 38 representative protein sequence of lineage 1–5 belongs to APMV-1. The tree is drawn to scale, with branch lengths measured in the number of substitutions per site. Evolutionary analyses were conducted in MEGA6. Malaysian isolates MB128/04, MB076/05, IBS002/11, and IBS005/11 presented in this study are marked with a triangle (Δ) and IBS025/13 with a circle (◦).

Genomic information is the basis used by the two currently available methods in NDV classification, where one is to identify strains by lineages and the other by classes/genotypes. The phylogenetic and pairwise sequence comparison data presented in this study is based on the lineage classification proposed by Aldous et al. (2003) and Munir et al. (2012). According to the phylogenetic analysis of partial F gene, all the isolates clustered with lineage 5 (Figure 1). Moreover, in line with the PASC analysis, the isolates also have the highest percentage identity (PI) to NDV strains belonging to lineage 5. The NDV isolates MB128/04 and MB076/05 isolated in 2004 and 2005, respectively, showed the highest PI of 92.41% to lineage 5 (Table 6). Meanwhile, PASC analysis of the genetic similarity among sub-lineage under lineage 5 indicated that the isolates showed the highest PI to sub-lineage 5a. However, except for IBS025/13, the genomic divergence between the lineage 5a and the sequenced viruses isolated during the period from 2004 to 2011 is increasing (Table 7). The phylogenetic tree was drawn based on nucleotide sequence of complete genome sequences, and it shows the clustering of the isolates with lineage 5 (Figure 2A). The robustness of the genetic groupings and topology of the phylogenetic trees were also confirmed by the findings obtained from the PASC analysis of the complete genome as well as F, HN, M, and L protein sequences of MB128/04, MB076/05, IBS002/11 and IBS005/11 isolates, respectively, whilst PASC analysis of NP and P protein sequences of IBS025/13 isolate showed the highest percentage identity to lineage 2 viruses (Table 8). Furthermore, maximum likelihood phylogenetic trees constructed based on NP and P proteins of IBS025/13 isolate indicated that those proteins were clustered with lineage 2 viruses and substantiated the results obtained from PASC analysis (Figure 2B).

**Table 6:** **Pairwise sequence comparison of the partial F gene sequences of Malaysian isolates based on different lineages**.

**NDV Isolate**	**Percentage identity (%)**				
	**1**	**2**	**3**	**4**	**5**
MB128/04	83.74	81.16	87.42	89.09	92.41
MB076/05	83.74	81.16	87.42	89.09	92.41
IBS002/11	81.48	79.84	84.65	85.81	88.42
IBS005/11	81.48	79.84	84.65	85.81	88.42
IBS025/13	83.57	80.66	86.50	87.87	91.32

**Table 7:** **PASC analysis of the partial F gene sequences of Malaysian isolates based on lineage 5**.

**NDV isolate**	**Percentage identity (%)**								
	**5a**	**5b**	**5c**	**5d**	**5e**	**5f**	**5g**	**5h**	**5i**
MB128/04	96.22	92.23	93.74	93.51	93.16	85.79	86.33	83.56	86.33
MB076/05	96.22	92.23	93.74	93.51	93.16	85.79	86.33	83.56	86.33
IBS002/11	91.51	87.57	90.88	90.03	88.67	81.77	82.31	79.54	83.51
IBS005/11	91.51	87.57	90.88	90.03	88.67	81.77	82.31	79.54	83.51
IBS025/13	94.82	91.37	92.58	92.6	91.89	84.45	84.46	81.68	85.66

**Table 8:** **Estimates of the evolutionary distance of NP, P, F, HN, M, L amino acid and complete genome nucleotides sequences between Malaysian NDV isolates and each lineage**.

**Gene**	**NDV isolate**	**Lineage 1**	**Lineage 2**	**Lineage 3a**	**Lineage 3b**	**Lineage 3c**	**Lineage 3d**	**Lineage 4**	**Lineage 5**
NP	MB128/4	5.51	7.72	5.68	4.61	4.07	4.03	4.31	2.96
	MB076/5	5.51	7.72	5.68	4.61	4.07	4.03	4.31	2.96
	IBS002/11	6.68	9.28	7.21	5.54	4.5	4.03	5.21	3.22
	IBS005/11	6.9	9.51	7.43	5.76	4.71	4.25	5.34	3.43
	IBS025/13	3.67	0.65	7.21	7.65	7.10	8.02	8.76	8.08
P	MB128/4	15.65	17.17	14.43	13.16	13.16	12.41	13.01	9.75
	MB076/5	15.65	17.17	14.43	13.16	13.16	12.41	13.01	9.75
	IBS002/11	17.62	18.52	15.7	14.68	14.68	14.18	14.43	9.82
	IBS005/11	17.62	18.52	15.7	14.68	15.06	14.68	14.94	10.23
	IBS025/13	9.16	2.62	11.90	12.83	16.08	14.26	16.61	16.46
M	MB128/4	9.29	12.64	10.99	9.98	10.30	6.50	5.60	4.26
	MB076/5	9.29	12.64	10.99	9.98	10.30	6.50	5.60	4.26
	IBS002/11	7.69	11.26	9.89	9.89	10.30	5.40	5.27	4.04
	IBS005/11	7.42	10.99	9.62	9.62	10.03	5.13	4.73	3.49
	IBS025/13	8.46	11.81	10.71	9.62	11.13	6.50	5.60	4.42
F	MB128/4	12.38	14.59	12.33	11.53	10.59	9.03	8.65	5.66
	MB076/5	12.38	14.59	12.33	11.53	10.59	9.03	8.65	5.66
	IBS002/11	14.03	15.68	13.42	12.84	11.88	10.69	10.67	6.58
	IBS005/11	14.21	15.68	13.6	13.06	11.85	10.87	10.95	6.76
	IBS025/13	13.19	15.62	13.06	12.90	12.06	10.43	10.17	6.29
HN	MB128/4	9.95	12.26	10.68	11.79	8.41	7.53	7.64	4.54
	MB076/5	9.95	12.26	10.68	11.79	8.41	7.53	7.64	5.15
	IBS002/11	10.68	13.34	11.21	12.96	9.19	8.06	8.72	8.72
	IBS005/11	10.19	12.81	10.68	12.43	9.02	7.76	8.44	5.81
	IBS025/13	10.61	13.11	11.91	12.84	9.63	8.41	8.65	6.01
L	MB128/4	5.17	6.54	5.04	4.52	4.11	4.18	4.21	2.44
	MB076/5	5.17	6.54	5.04	4.52	4.11	4.18	4.21	2.44
	IBS002/11	5.25	6.55	5.4	4.93	4.31	4.18	4.28	2.56
	IBS005/11	5.2	6.51	5.27	4.77	4.18	4.04	4.16	2.39
	IBS025/13	5.12	6.56	5.13	4.65	4.15	4.25	4.41	2.55
Complete	MB128/4	17.93	19.79	16.14	15.21	12.88	13.09	11.98	7.87
	MB076/5	17.90	19.83	16.10	15.17	12.84	13.04	11.92	7.82
	IBS002/11	18.85	20.56	16.78	16.27	14.28	14.17	13.45	8.62
	IBS005/11	18.82	20.37	16.72	16.16	14.26	14.12	13.35	8.49
	IBS025/13	16.73	14.62	16.11	15.92	15.03	14.99	14.61	10.09

Underlined values show the minimum evolutionary distances between each lineage and Malaysian NDV isolates. Using MEGA6 program, the data was generated and values indicates% nucleotide and amino acids distance respectively.

### Recombination analysis of NDVs using simplot and RDP

**Figure 3:**
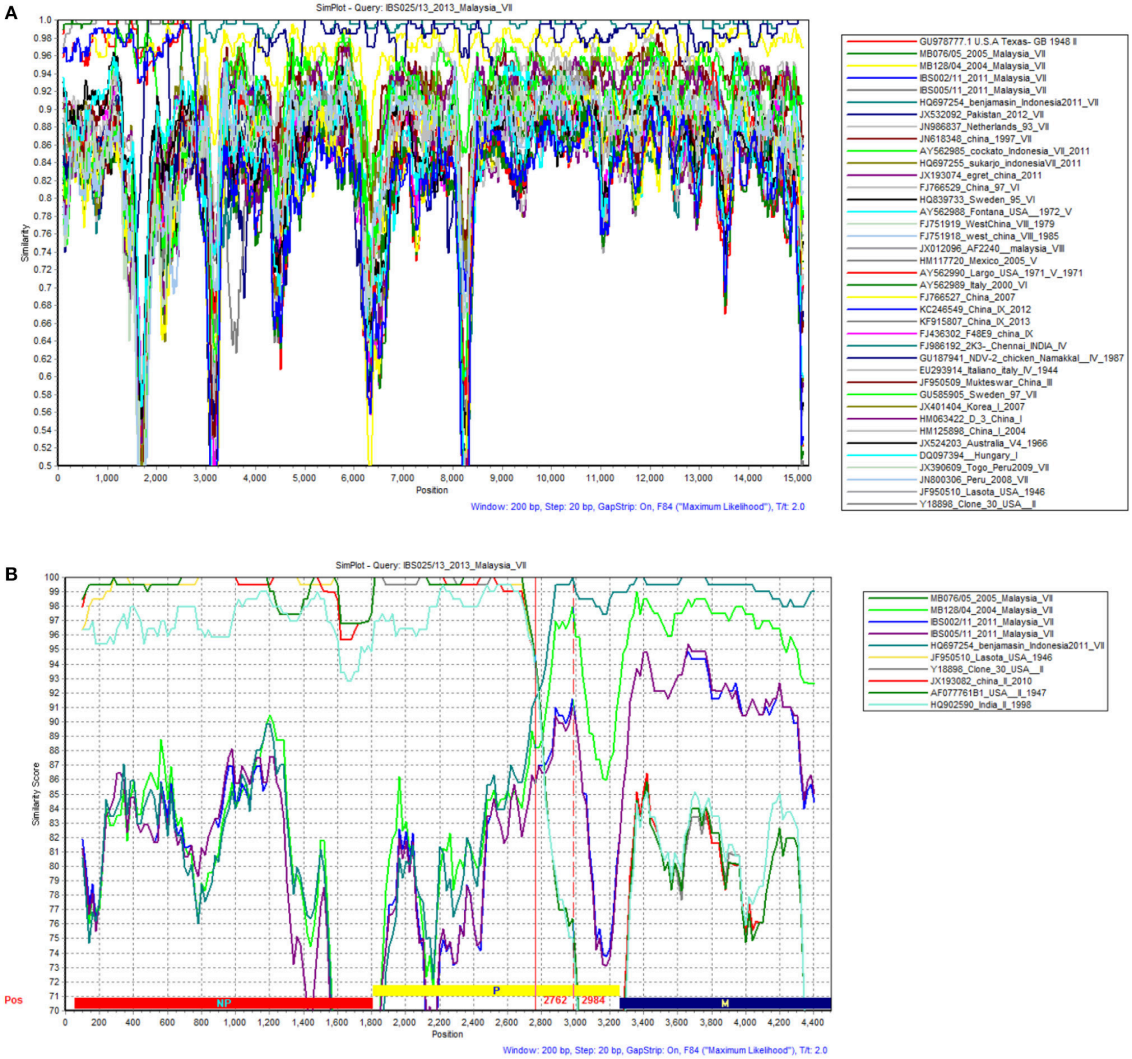
**Results from SimPlot Analysis of IBS025/13**. The evidence for recombination of strain IBS025/13. **(A)** The similarity of the complete genome of IBS025/13 from SimPlot analysis with 38 representative complete genomes belonged to APMV-1. Standard similarity plot constructed using all sites of the complete genome within a sliding window of 200 bp wide centered on the position plotted with a step size between plots of 20 bp. The y-axis gives the percentage of identity. **(B)** The similarity of the first 1/3 genome representing NP, P, and M gene of IBS025/13 from SimPlot analysis with most similar nucleotide sequences belong to APMV-1. Standard similarity plot constructed using all sites of the first 1/3 genome with a window size of 200 bp and a step size of 20 bp. The y-axis gives the percentage of identity. Red vertical lines indicate the possible breakpoints.

**Figure 4:**
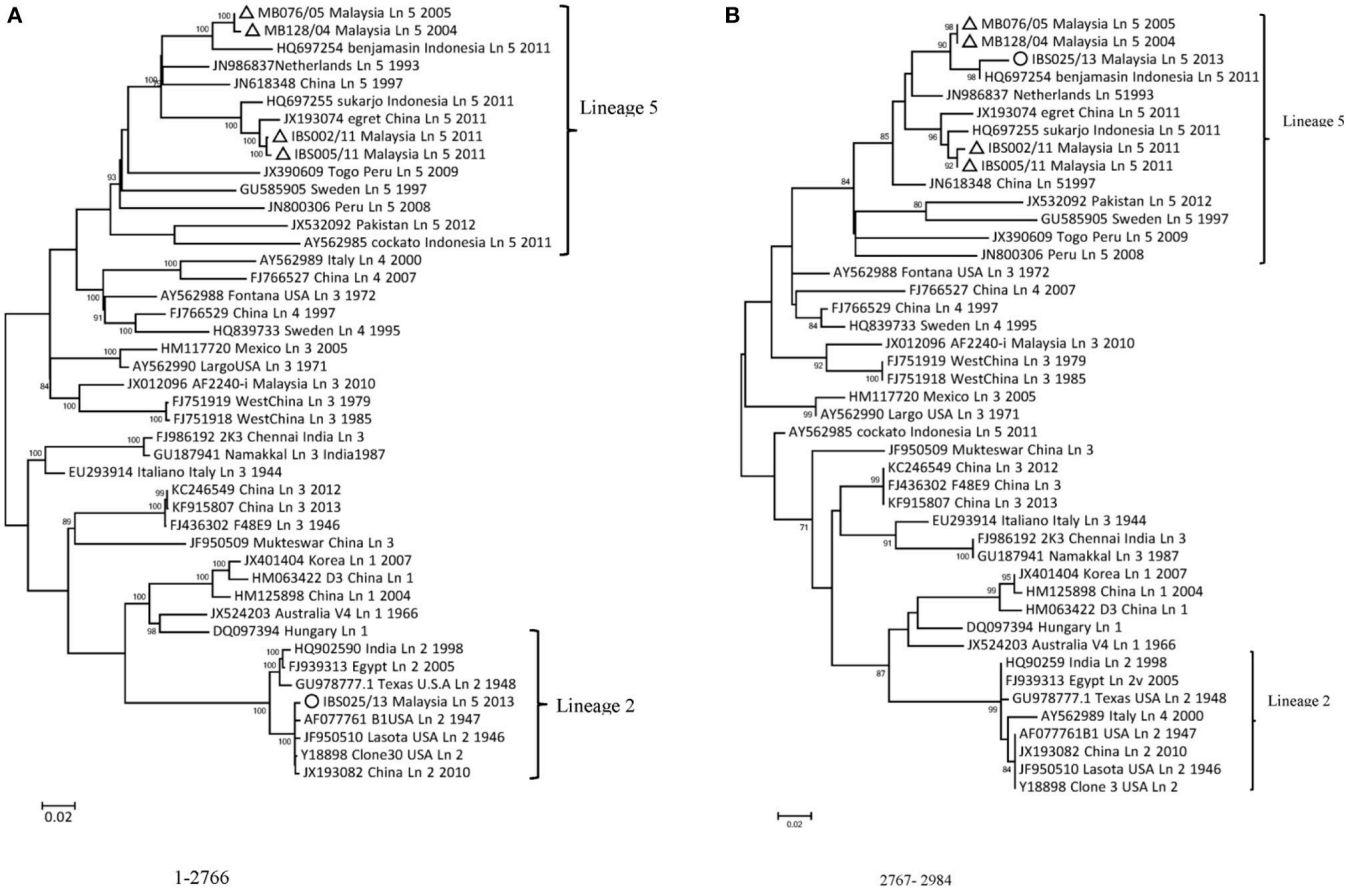
**Neighbor-joining phylogenies inferred for the two regions (A,B)** delimited by the breakpoints. The percentage of replicate trees in which the associated taxa clustered together in the bootstrap test (1000 replicates) is shown next to the branches (only N70% is shown). The tree is drawn to a scale, with branch lengths in the same units as those of the evolutionary distances used to infer the phylogenetic tree. The Maximum Composite Likelihood model as described in Methods, and are in the units of the number of base substitutions per site was used to compute the evolutionary distances. Codon positions included were 1st + 2nd + 3rd + non-coding. All the positions that contained gaps and missing data were removed completely from the dataset (complete deletion option). Phylogenetic analyses were conducted in MEGA6. Malaysian isolates presented in this study are marked with a triangle (Δ) and the putative mosaic is indicated with a circle (◦).

A standard similarity plot (Simplot) (Ray, 2003) was used to analyze the possible events of recombination in the sequenced isolates with representative isolates of lineages 1–5 obtained from GenBank. Since the analysis revealed that NP and P gene regions of isolate IBS025/13 have the highest genomic similarity toward lineage 2 viruses over lineage 5 (Figures 3A,B), we also investigated the first 1/3 of NDV genome alignment to identify potential breakpoints by using recombinant detection program (RDP) (Martin et al., 2005). Possible breakpoints at nucleotide position 1–2766 and 2767–2984 were detected by *p*-value equal to RDP (8.418E-4), Geneconv (1.617E-3), BootScan (8.349E-4), MaxChi (1.612E-1) and SiScan (2.509E-10). The most convincing evolutionary evidence for recombination was the occurrence of incongruent phylogenetic trees (Zhang et al., 2010). Maximum composite likelihood phylogenic trees were constructed in both break points using MEGA 6 software with 1000 bootstrap values according to the best model selected on lowest BIC. The results confirmed clustering of IBS025/13 isolate with lineage 2 NDVs at breakpoint 1–2766 and breakpoint 2767–2984 with lineage 5 NDVs (Figures 4A,B). Further evidence on the event of recombination in NDV was provided by sequence comparisons within the species-defining clusters (Han et al., 2008). Nucleotide fragment of 1–2766 of IBS025/13 was used as a query in BLAST in the GenBank, which showed the highest sequence identity of 99% to Lasota (AF077761), B1 (AF309418), Clone 30 (Y18898) lineage 2 NDV vaccine strains (data not shown). Sequence analysis confirmed the NP gene of IBS025/13 from nucleotide position 55–1801, which include both the 3′ and 5′ untranslated regions (UTR) were derived from lineage 2 isolates (Table 4). In addition, the deletion of 6 nts at the UTR region of NP gene revealed similar genome length to lineage 2 NP genes. Meanwhile, the entire 3′ UTR of P gene (1804–1886) and two-thirds of P gene from position 1887–2776 of IBS025/13 also resemble lineage 2 isolates (Table 4).

## Discussion

In 2013, more than 3000 ND outbreaks were reported from Asian countries to the World Organization for Animal Health (WAHID, 2015). Characterizing and understanding the molecular epidemiology of the currently circulating NDV strains in the world, is essential for the controlling and preventing ND outbreaks (Miller et al., 2010). The current approach used only five sets of primers to cover the whole genome of NDV, and the protocol could support the multiplexing of 94 samples by tagging unique combination of indexes during a single sequencing run with a fast turn-around time, which dramatically reduced the cost and laborious work needed to be conducted in complete genome sequencing. Since adopted method used a unique combination of indexes to each NDV sample during multiplexing and raw sequenced data was filtered according to the tagged indexes, generated sequences are originated purely from the NDV isolates obtained from outbreaks. Moreover, NGS based on Ion Torrent Personal Genome Machine (PGM, Life technologies), has been used to sequence the complete genome of avian paramyxoviruses type 4 (Wang et al., 2013).

Genome lengths of the MB128/04, MB076/05, IBS002/11 and IBD005/11 isolates were similar to the genome length of genotype V–VIII. and IX in class II APMV-1. Studies have shown that the genome length of these genotypes have evolved by an insertion of 6 nts at the nucleotide position of 1647 in the 5′ non-coding region (NCR) of the NP gene of early genotypes (I–IV), which have genome length of 15,186 nts (Czeglédi et al., 2006). Interestingly, genome length of IBS025/13 NDV isolated in 2013 was 15,186 nts, similar to the genome length of early NDV genotypes (I–IV) isolated during 1930–1960s (Czeglédi et al., 2006). In this isolate, deletion of 6 nts at the same position of 5′ NCR of the NP gene was observed subsequently confirmed by targeted region amplification and sequencing using primer walking technology. In addition, the NP and P gene regions of IBS025/13 were sequenced thrice using Sanger sequencing to confirm the recombinant events.

The OIE recognizes the ICPI as a test which can be used to assess the pathogenicity of NDV. An NDV strain with an ICPI ≥ 0.7 is identified as a virulent or “notifiable” to the OIE. The cleavage site motif ^112^R/K-R-Q-R/K-R↓F^117^ is cleavable by a wide range of proteases that subsequently cause systemic infection (Panda et al., 2004; Maminiaina et al., 2010; Miller et al., 2010). The cleavage site motif of the isolates also demonstrated the sequences of velogenic strains and these findings confirm the velogenic nature of the Malaysian NDV isolates (Table 5) reported in this study.

Previously, Munir et al. (2012) have indicated that NDV sub-lineages under lineage 5 can be identified with cut-off PI at 95% based on partial F gene sequences. In that study, viruses in lineage 5 can be further divided into 9 sub-lineages, from 5a to 5i with previously characterized NDV isolates including recent isolates reported from African countries and Pakistan. Based on the above study, MB128/04 and MB076/05 can be placed in the sub-lineage 5a. One of the salient findings of this study is, although IBS002/11 and IBS005/11 that were isolated in 2011 have the highest PI-value to the sub-lineage 5a, the PI-value was <95%. The findings illustrate the evolutionary pattern observed in NDV isolates from 2004 to 2011 and urged a reviewing of the current sub-lineage cut off point of lineage 5 in NDV classification or placing NDV isolates IBS002/11 and IBS005/11 under a new sub-lineage of lineage 5. Furthermore, the findings of this study support a reviewing of the current nomenclature of NDV as proposed by Miller et al. (2010) and Munir et al. (2012). The phylogenetic tree drawn based on complete nucleotide sequences of isolates shows the clustering of the isolates with lineage 5 and these findings agreed with Miller et al. (2010), who propounded that currently, the predominant circulating NDV isolates in the Asian region are of lineage 5.

In comparison to the traditional NDV classification method by partial F gene, the whole genome analysis is advantageous for the identification of recombination events. Other previous studies have reported the evidence of recombination in F gene sequences between genotype II and genotype VII, poultry and ostrich NDVs (Yin et al., 2011). Moreover, Chong et al. (2010) and Han et al. (2008) have reported recombinants of NDVs that involved multiple genotypes. The results of the current study present the first natural recombination event detected between lineage 2 and 5 following isolation and sequencing of virus from an ND outbreak in chicken.

Vaccination against NDV has been described as the most effective prevention strategy seconded to strict bio-security measures in modern poultry farming where live and killed vaccines are widely used throughout various countries in the world. Despite intensive vaccination, the occurrence of NDV outbreaks in endemic countries in Asia, Africa, and Central America is puzzling (Czeglédi et al., 2006). High-density rearing in modern poultry farming, which enhances close animal-to-animal contact, favoring transmission of virulent viruses over milder forms and selective immune pressure exerted by improper vaccination, can affect the evolution of circulating virulent viruses (Miller et al., 2010; Zenglei and Liu, 2015). Additionally, improper vaccination strategy, immune suppression and presence of variant NDV strains have been implicated as the main underlying factors of poor vaccine efficacy against ND (Okoye and Shoyinka, 1983; Yu et al., 2001; Cho et al., 2007; Dortmans et al., 2012; Perozo et al., 2012).

Based on bioinformatics analysis, it has been reported that vaccination with live attenuated viruses altered the evolution of NDV by sustaining a large effective population size of a vaccine-related genotype, allowing for co-infection and recombination of vaccine and wild-type strains (Chong et al., 2010). In that study, recombination events in NP gene of Cockatoo/14698 Indonesia (AY562985) and F gene of Layer/SRZ03 China (EU167540) of genotype VII isolate with genotype I isolates were identified by maximum likelihood trees and RDP3 program (Chong et al., 2010). The current study provides the evidence of recombination in NDV between vaccine lineage and the circulating lineage 5 following an isolation and sequencing. Since live attenuated vaccines are being carried out in most of the NDV-endemic countries, the likelihood of recombination between vaccine and field strains is relatively high. The magnitude of occurrence and underlying molecular mechanisms of natural recombination in NDV is not well documented. Many research groups have documented natural recombination events for NDV (Han et al., 2008; Qin et al., 2008; Chong et al., 2010; Zhang et al., 2010; Yin et al., 2011). However, it has also been suggested that the recombination between different NDV strains is a rare event (Afonso, 2008) and that apparent genetic recombination in NDV may be an artifact (artificial recombination) (Song et al., 2011). Nevertheless, evidence of recombination between wild type and vaccine strains of NDV is relatively under-reported compared to other RNA viruses including avian influenza viruses (Webster et al., 1974), infectious bursal disease virus (Hon et al., 2008) and infectious bronchitis virus (Cavanagh et al., 1992).

Taken together, this study has described the application of NGS-based technology for complete genome sequencing of genotype VII NDV isolates. The protocol was able to generate consensus-level full genome sequence of five virulent NDVs obtained from outbreaks during 2004–2013 in Malaysia. The in-depth studies conducted on the different genotype VII NDV demonstrated the increase in the evolutionary distance of F and HN proteins of circulating lineage 5 NDVs against lineage 2. Furthermore, this study provides the evidence of recombination between the vaccine lineage and circulating virus, which warrants the importance of continuous investigations on the genome-wide study of NDV diversity.

## Author contributions

Conceived and designed the experiments: AO, DS, ST, and SY. Performed the experiments: DS and KM. Analyzed the data: DS, AO, ST, and MM. Contributed reagents/materials/analysis tools: AO and AI. Wrote the manuscript: DS. Critical revision: AO and MM.

## Funding

This work was supported by an Institute of Bioscience, Higher Institution Centre of Excellence grant (IBS HICoE 6369101) from the Ministry of Education, Government of Malaysia, Universiti Putra Malaysia Grant No. P-IPB/2013/9425700, and Biotechnology and Biological Sciences Research Council (BBSRC) through Institute Strategic Program Grant (BB/J004448/1).

### Conflict of interest statement

The authors declare that the research was conducted in the absence of any commercial or financial relationships that could be construed as a potential conflict of interest.
